# The Relationship between Cognitive Impairment and Coronary Artery Disease in Middle-aged Adults

**DOI:** 10.7759/cureus.6724

**Published:** 2020-01-21

**Authors:** Nasser T Balbaid, Ahmad Al-Dawalibi, Abdullah M Khattab, Fahad Al-Saqr, Abdulrahman AbuSittah, Sami Alqarni, Syed Habib, Muhammad Iqbal, Shahid Bashir

**Affiliations:** 1 Physiology, College of Medicine, King Saud University, Riyadh, SAU; 2 Neurophysiology, Neuroscience Center, King Fahad Specialist Hospital, Dammam, SAU

**Keywords:** coronary artery disease, cognitive impairment, cognitive function

## Abstract

Background

Cognitive impairment is a phenomenon that appears late in many diseases. Many clinicians do not seriously consider cognitive impairment until there has been significant deterioration over time. Cognitive function can be assessed using the Cambridge Neuropsychological Test Automated Battery (CANTAB).

Methods

Using an observational case-control study design, we examined the relationship between coronary artery disease (CAD) and cognitive impairment. Participants (57 patients with CAD and 60 healthy controls; age: 30-60 years) were recruited and sampled using a non-probability quota sampling technique. Self-administered questionnaires were used to collect data about participants' demographic information. Blood chemistry samples were obtained to evaluate patients for CAD. Mini-Mental State Examination (MMSE) and CANTAB using three subsets including intra-extra dimensional set shift, spatial span (SSP), and pattern recognition memory (PRM) were used to assess participants' cognitive function.

Results

The SSP and PRM were significantly lower in patients with CAD as compared with healthy controls. There were significant relationships of PRM with creatine kinase- muscle/brain, aspartate aminotransferase, and total cholesterol. On the other hand, SSP was found to have a significant relationship with triglycerides.

Conclusion

There is cognitive impairment in CAD patients that needs to be assessed for early interventions to maintain cognitive functions.

## Introduction

Cardiovascular diseases (CVDs) are a group of diseases that affect the heart or blood vessels [1]. Most heart diseases present in the form of coronary artery disease (CAD), which is a predominant cause of mortality in patients with heart disease [2]. Though death due to CVD is declining, CVD is still the leading cause of non-communicable disease (NCD) mortality and morbidity worldwide [3]. CVD in the Kingdom of Saudi Arabia (KSA) is a cause of the majority of deaths, exceeding untreatable fatal diseases such as cancer [4]. According to a World Health Organization (WHO) report, 644 in 100,000 people aged 30-70 years died due to NCD, 62% (401 per 644) of which were due to CVD and diabetes in KSA [4]. This raises a huge concern for health care institutions to develop health protocols to detect heart problems before they reach an advanced stage. CAD or ischemic heart disease (IHD) is a common form of CVD that is caused by an obstruction or disruption of coronary blood flow usually due to an advanced atherosclerotic lesion that will eventually lead to a disorder known as myocardial infarction or ischemia [5]. The prevalence of CAD in KSA is 5.5% [6]. Recently, it has been shown that there is a possible correlation between CAD and impaired cognitive function, which affects the mental capacity and health status of CAD patients [7]. Recent studies have reported a marked decrease in regional brain volume in patients with CAD accompanied by significant cerebral atrophy, hypoperfusion, and white matter disease in the brains of older adults with an increased risk of vascular disease [7-8]. Although these observations suggest a possible link between CAD and cognitive decline or neurological dysfunction, the interaction of age, vascular disease, and cerebrovascular dysfunction are complex and not well understood.

Depression is the most common comorbid psychiatric disorder in patients with chronic illness such as CAD and chronic obstructive pulmonary disease, with rates consistently found to be two to three times higher than those in the general population [9]. The prevalence of depression is twice as higher in women than in men [10]. About 17-27% of patients with CAD have major depression [11]. Although the presence of a relationship between depression and cardiac disease is obvious, the mechanisms underlying this remain unclear.

Some studies have hypothesized underlying mechanisms such as neuroendocrine dysfunction, while also suggesting other important factors such as smoking history, sedentary lifestyle, and a delay in seeking treatment [12-13]. According to the WHO, overweight and obesity are defined as excessive fat accumulation that may impair body function and ultimately health status. In 2014, more than 1.9 billion adults aged 18 years and older were overweight, with over 600 million of these adults being obese [14]. A recent study among university students in KSA has reported a 36.8% prevalence of obesity and overweight [15]. Other studies also found that obesity is associated with an increased risk of CVD and type 2 diabetes [16]. Some suggest that obese patients have a higher risk of morbidity and mortality [17]. Furthermore, obesity is the most prevalent risk factor for CAD [18]. Some studies have shown that elevated levels of adipokines are linked to increased body mass index (BMI) [19]. Adipose tissue secretes hormones, peptides, and other molecules that may potentially act as pro-atherogenic markers [20].

This study was conducted to evaluate the cognitive impairment hypothesis in patients with CAD and aims to provide better preventive strategies in the near future. This study includes mental status evaluation along with lab investigations and imaging studies to elucidate the relationship between cognitive impairment and CAD severity in middle-aged patients in KSA.

## Materials and methods

Participants

Study Design, Setting, and Sampling Technique

An observational case-control study was performed at the Departments of Physiology and Cardiac Sciences, College of Medicine, King Khalid University Hospital, Riyadh, KSA. A non-probability quota sampling technique was used to recruit our participants (57 patients with CAD and 60 healthy controls). Participants were included or excluded as per the selection criteria.

Inclusion and Exclusion Criteria

Inclusion in the patient group required participants to be diagnosed with CAD based on symptoms, medical history, and risk factors. Participants had to be aged between 30 and 60 years. Patients with any history of neurological or psychological disorders such as Alzheimer’s disease, depression, cognitive impairment, anxiety, transient ischemic attack, and stroke were excluded. Participants were also excluded if they presented with any disorders that may affect the brain, such as thyroid disorders, chronic liver disease, chronic kidney disease, and acute diabetic states such as diabetic ketoacidosis. The participants in the healthy control group were interviewed to confirm that there were free of cardiovascular illness, medications, and significant underlying neurological or psychiatric illness, and had no history of head injury or drug/alcohol abuse that would potentially affect their cognitive function.

Instruments used

Assessment of Participants' Baseline Information

Self-administered questionnaires were used to collect data about participants' demographic information. Blood chemistry samples were also obtained to evaluate patients for CAD. Venous blood samples were analyzed for brain natriuretic peptide, creatine kinase-muscle/brain (CKMB), alanine aminotransferase, aspartate aminotransferase (AST), total cholesterol (TC), low-density lipoprotein, high-density lipoprotein, and triglycerides (TGs).

Mini-Mental State Examination

The Mini-Mental State Examination (MMSE) is one of the most widely used tools for quantitative assessment of cognitive function. The test consists of 11 questions assessing various cognitive functions, including two questions concerning orientation, one concerning registration, one concerning memory, five concerning language, one concerning attention and calculation, and one concerning visual construction [5]. The MMSE takes 5-10 minutes to administer, making it a practical tool for research purposes. The test has a maximum score of 30, with a score below 23 being indicative of cognitive impairment. The standard MMSE is in English and was used with English-speaking participants [5]. For Arabic speakers, a translated version of the MMSE was used; this version was introduced in 1999 by a group of Saudi researchers [6].

Cambridge Neuropsychological Test Automated Battery

The Cambridge Neuropsychological Test Automated Battery (CANTAB) test was used utilizing the following three subtests [8]:

1. Intra-extra dimensional set shift (IED): IED set shift is a test of rule acquisition and reversal. Stimuli consisting of two potential dimensions, color-filled shapes and white lines, were used in the test. Simple stimuli consisted of just one of these dimensions, whereas compound stimuli consisted of both, namely white lines overlying color-filled shapes. The participants started by seeing two simple color-filled shapes. They then had to learn which one was correct by selecting a stimulus and then receiving a feedback, which allows the participant to know which stimulus is correct. After six correct responses, the stimuli and/or rules are changed. These shifts are initially intra-dimensional (e.g. color-filled shapes remain the only relevant dimension) and later extra-dimensional (white lines become the only relevant dimension). Participants progress through the test by satisfying a set criterion of learning at each stage (six consecutive correct responses). If at any stage the participant fails to reach this criterion after 50 trials, the test terminates.

2. Spatial span (SSP): the SSP assesses working memory capacity using white squares that are displayed on a screen, some of which briefly change color in a variable sequence. The participant must then select the boxes which changed color in the same order in which they were displayed by the computer (for the forward variant) or in the reverse order (for the backward variant). The number of boxes in the sequence increases from two at the start of the test to nine at the end of it. The sequence and colors are varied throughout the test.

3. Pattern recognition memory (PRM): the PRM is a measure of visual PRM tested in a two-choice forced discrimination paradigm. The participants were presented with a series of visual patterns, one at a time, in the middle of the screen. These patterns were designed such that they cannot be easily given verbal labels. In the recognition phase, the participants were required to choose between a pattern they had been shown and a novel pattern. This was repeated with new patterns. The second recognition phase was given immediately or after a delay.

Statistical analysis

Data were analyzed using SPSS for Windows, Version 21.0 (IBM Corp., Armonk, NY). Categorical data are expressed as absolute numbers and percentages. Numeric data are summarized as mean, median, standard deviation (SD), and range. Categorical variables were compared using the chi-square test. Student’s t-test was used for normally distributed data, and the Mann-Whitney U test for non-normally distributed data. Spearman’s rank order and Pearson correlations were used to assess the association between variables. Two-tailed P-values less than 0.05 were considered statistically significant.

## Results

Sample characteristics

The patient group (n=57) had a mean age of 48.63±8.42 years, whereas the control group (n=60) had a mean age of 46.37±7.46 years. The difference in mean age between the two groups was not statistically significant (p=.130). There was a significant difference in MMSE score among two groups (p=.043). The demographic data of the participants are summarized in Table 1.

**Table 1:** Characteristics of study patients with coronary artery disease and healthy control.

Variables	Patients (n=57)	Healthy Control (n=60)	P-Value
Age	48.63±8.42	46.37±7.46	.130
Body mass index	28.12±4.62	28.24±4.51	.910
Mini-Mental State Examination	27.46±2.12	28.26±1.54	.043

Cognitive function

There was no significant difference in IED total between the patient and control groups (p=.293) (Table 2). Additionally, there was no significant difference between the two groups in the number of IED stages completed (p=.413). However, there was a significant difference in PRM (p=.002) and SSP (p=.000) results between patients and controls.

**Table 2:** Comparison of cognitive functions between coronary artery disease patients and healthy control. SD, standard deviation; IED, Intra-extra dimensional set shift; PRM, pattern recognition memory; SSP, spatial span

Variables	Patients, Mean ± SD	Healthy Control, Mean ± SD	P-Value
IED total errors (adjusted)	23.50±41.36	14.97±20.06	.293
IED stages completed	8.55±1.82	8.84±.884	.413
PRM percent correct	71.04±13.29	81.53±13.95	.002
SSP length	4.76±1.47	6.03±1.77	.000

Table 3 shows the significant relationships of PRM with CKMB (r=.526; p=0.025 ), AST (r=.371; p=0.031), and TC (r=.420; p=0.019). On the other hand, SSP was found to be significantly associated with TG (r=.421; p=0.036).

**Table 3:** Correlation of cognitive task and blood marker in coronary artery disease patients. BNP, brain natriuretic peptide; CKMB, creatine kinase-muscle/brain; ALT, aminotransferase; AST, aspartate aminotransferase; LDH, lactate dehydrogenase; TC, total cholesterol; HDL, high-density lipoprotein; LDL, low-density lipoprotein; TG, triglycerides *Correlation is significant at the 0.05 level

	IED Total Errors (Adjusted)	IED Stages Completed	PRM Percent Correct	SSP Length
BNP	-.082	.400	-.284	.053
CKMB	-.036	.159	.526^*^	-.040
ALT	.014	.211	.320	.199
AST	.228	-.035	.371^*^	-.005
LDH	-.108	.166	.072	.250
TC	.163	-.342	.420^*^	.264
HDL	.166	-.321	-.055	.037
LDL	.014	-.103	-.027	.009
TG	.033	-.337	.312	.421^*^

## Discussion

The prevalence of cognitive impairment is very high in CAD patients and is approximately 35% [21]. These figures are alarming and need further exploration of possible causes of cognitive decline in such cases. Similar to the results of our study, cognitive decline in memory pattern tested by PRM and SSP has been reported in CVD patients, which is aggravated after cardiac catheterization. The possible reasons could be ischemic insults that the brain receives during such events [22]. Another possible reason for cognitive decline in CVD patients may be the use of cardiovascular drug that can lead to cognitive decline. The new point raised in our study is that we assessed cognition through a standard computer software CANTAB (Cambridge Cognition Ltd., Cambridge, UK) [23-24].

In a recent report, a standardized battery of tests recommended by the National Institute of Neurological Disorders and Stroke-Canadian Stroke Network for the investigation of vascular cognitive impairment and dementia was used to assess cognitive performance. The results were similar to those of our study in that patients with CAD and endothelial dysfunction had poor verbal memory and that improvement in endothelial function during treatment was significantly associated with improvements in overall cognition and processing speed [25].

Another study reported that poorer peak oxygen uptake is associated with poorer cognition, particularly executive function, in patients with CAD independent of other cardiac risk factors. Cardiopulmonary fitness may be a protective factor for cognition in patients with CAD [26]. Our data indicate the importance of neurocognitive assessments in CVD patients and opens doors for the beneficial effects of lifestyle modifications and pharmacotherapy in CVD.

## Conclusions

The presence of cognitive impairment among CAD patients is an important problem and needs to be recognized to allow for interventions to maintain cognitive functions. Early memory assessment in patients could help the timely introduction of treatments, including memory training, and could be used as a tool for classifying symptoms indicating disease deterioration.
